# Controlled electron injection facilitated by nanoparticles for laser wakefield acceleration

**DOI:** 10.1038/s41598-018-34998-0

**Published:** 2018-11-16

**Authors:** Myung Hoon Cho, Vishwa Bandhu Pathak, Hyung Taek Kim, Chang Hee Nam

**Affiliations:** 1 Center for Relativistic Laser Science (CoReLS), Institute for Basic Science, Gwangju, 61005 Korea; 2 Advanced Photonics Research Institute (APRI), Gwangju Institute of Science and Technology, Gwangju, 61005 Korea; 3 Department of Physics and Photon Science, Gwangju Institute of Science and Technology, Gwangju, 61005 Korea

## Abstract

We propose a novel injection scheme for laser-driven wakefield acceleration in which controllable localized electron injection is obtained by inserting nanoparticles into a plasma medium. The nanoparticles provide a very confined electric field that triggers localized electron injection where nonlinear plasma waves are excited but not sufficient for background electrons self-injection. We present a theoretical model to describe the conditions and properties of the electron injection in the presence of nanoparticles. Multi-dimensional particle-in-cell (PIC) simulations demonstrate that the total charge of the injected electron beam can be controlled by the position, number, size, and density of the nanoparticles. The PIC simulation also indicates that a 5-GeV electron beam with an energy spread below 1% can be obtained with a 0.5-PW laser pulse by using the nanoparticle-assisted laser wakefield acceleration.

## Introduction

Laser wakefield acceleration (LWFA) is a promising method to realize compact high-energy electron accelerators because of its significant acceleration field beyond 1 GV/cm^1^. In the past decade, LWFA has progressed to achieve multi-GeV energies^2–5^, small energy spreads^6^, stable^7^, and high charge^8^ electron beams. In LWFA, an intense laser pulse propagating into a plasma expels electrons away from its axis, and the restoring force induced by charge separation leads to recursive electron motion that excites plasma waves. When an electron bunch is injected into the plasma wave, it is rapidly accelerated by the strong electric field in the plasma wave. The electron injection is one of the key features that determine the beam charge and energy spread of the accelerated beam. As electron injection relies mostly on the self-injection scheme triggered by the extreme nonlinear behavior of the plasma waves such as wave breaking^9,10^ and plasma wave expansion^11^, it is difficult to control the injected charge, energy spread, and injection position in LWFA. Furthermore, the electron self-injection can be a critical issue in high-repetition-rate LWFA as the available laser intensity is limited because of technological constraints^12,13^. To overcome the limitations of self-injection, various electron injection methods have been proposed using colliding laser pulses^14–17^, density gradients^7,18,19^, inner-shell ionization^16,20,21^, and external magnetic fields^22^. However, these methods suffer from issues such as disturbed acceleration process, difficulties in controlling the charge and energy spread, and poor injection position stability. Thus, a controllable and localized electron injection scheme without disturbing the acceleration process would be essential for improving the electron beam properties, which is still a challenge.

Electron injection is a serious issue that prevents higher electron energies from being achieved in LWFA. The electron energy can, in principle, be increased by applying a higher laser power to a plasma with lower densities and longer lengths. As the plasma density decreases, however, the electron injection threshold rises because of the higher phase velocity of the plasma wave^23^. Thus, the plasma density should be higher than the threshold for self-injection at a given laser power, which limits the possible acceleration length. This conflicting behavior between the electron injection and acceleration process restricts the enhancement of electron energy. Therefore, a novel electron injection scheme is desirable to improve the achievable electron energies beyond the limitations of current injection methods.

In this article, we propose a controllable electron injection scheme using nanoparticles inserted into a plasma medium. The nanoparticle is ionized by the leading edge of the laser pulse, and the electric field of the ionized nanoparticle assists the electron injection into the plasma wave. The scheme may require a high temporal contrast to produce the ionized nanoparticle core in a short time; otherwise, nanoparticles may explode before the nanoparticles meet the wakefield. Nanoparticles, produced by various techniques, can be delivered as a stream with a position accuracy of a few microns^24–26^. To demonstrate the electron injection assisted by nanoparticles, we performed two-dimensional (2D) and three-dimensional (3D) particle-in-cell (PIC) simulations using JoPIC^27^. The PIC simulations indicate that for the laser-plasma parameters that cannot trigger self-injection, a nanoparticle in the plasma leads to controlled electron injection. The simulations also indicate that the beam-charge can be controlled by adjusting the nanoparticle parameters such as material density, number, and position.

**Figure 1:**
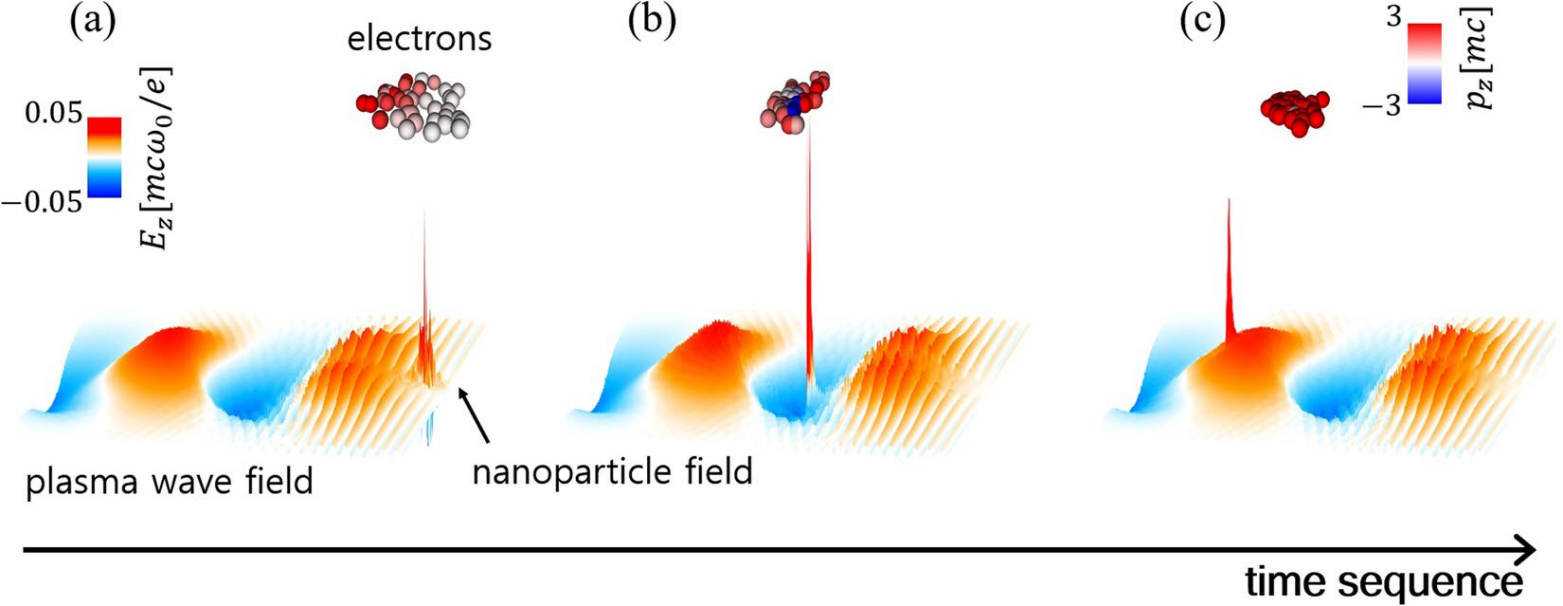
The time sequence of the electron injection process under the influence of a nanoparticle. (**a**) Nanoparticle field starts to grow and attracts vicinal electrons. (**b**) The attracted electrons obtain additional momentum during the interaction with the nanoparticle field and the plasma wakefield. (**c**) The electrons obtaining additional momentum are injected into the plasma wave.

A nanoparticle in plasma can induce a localized injection by supplying additional longitudinal momentum to the electrons attracted by the nanoparticle field. The electron injection process is illustrated in Fig. 1, which shows the evolution of the longitudinal electron momentum, laser wakefield, and nanoparticle field. In Fig. 1a and b, the charge separation by the ponderomotive force gradually increases the nanoparticle field and attracts adjacent electrons. The attracted electrons experience the nanoparticle potential asymmetrically because of the time-evolving nanoparticle field or the asymmetric electron motion around the nanoparticle, which leads to a net momentum gain. When the attracted electrons reach the trailing part of the wakefield, as shown in Fig. 1c, the electrons with a sufficient momentum are injected into the plasma wave.

## Results

### Proof-of-principle 3D PIC simulation

**Figure 2:**
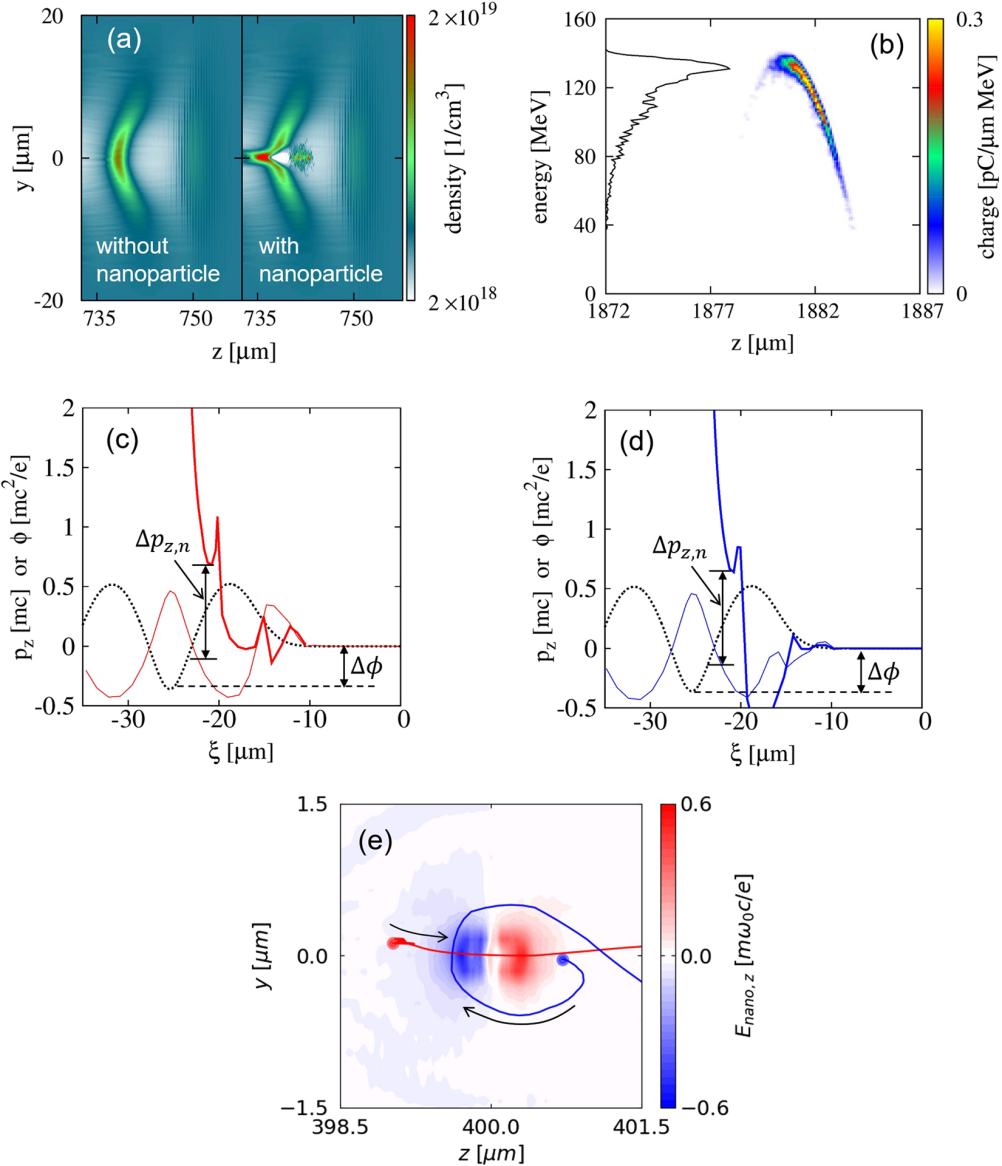
Proof-of-principle 3D PIC simulation showing the results with and without a nanoparticle on a laser axis (**a**) Electron density distribution in the z-y plane. (**b**) Energy spectrum and phase-space electron density when the electron beam reaches its maximum energy. (**c**,**d**) Typical electron trajectories in phase space with (thick solid line) and without (thin solid line) a nanoparticle. The plasma wave potential is drawn as the black dashed line. (e) Spatial distribution of the z-component of the nanoparticle field, and two trajectories of injected electrons, shown in (**c**) and (**d**), in z-y plane. The red dot and line indicate the trajectory of an electron starting from the left side of the nanoparticle, and the blues show that from the right side. The laser pulse is propagating from left to right.

For a proof-of-principle study on the nanoparticle-assisted electron injection, 3D PIC simulations were carried out. In the simulations, the laser intensity and plasma density are sufficiently high to excite nonlinear plasma waves but not enough for electrons self-injection i.e the wave-breaking conditions are not met without external influence. The laser pulse also does not significantly modified during the propagation through the plasma medium; *a*_0_ of the laser pulse increases up to 1.45 (initially 1.0), then decreases slowly to 0.7 when the electrons reached the maximum energy at *z* = 2 *mm*. Two cases, with and without a nanoparticle, were tested. Details of the simulation parameters are given in the method section. Figure 2a shows a comparison of the electron density distributions for the two cases at 750 μm from the plasma entrance. It demonstrates that electron injection is clearly induced by the insertion of a nanoparticle. The obtained electron bunch reaches a maximum energy of 130 MeV with a 5.7-pC charge after propagating 1.8 mm, as shown in Fig. 2b. The maximum energy is almost identical to the theoretical estimation 128 MeV in the linear regime^28,29^, indicating that the acceleration process is not disturbed by the insertion of the nanoparticle. Note that the laser profile is not significantly modified by the nanoparticle, due to its much smaller size than the laser wavelength. Thus, the simulation result clearly indicates the effectiveness of the nanoparticle-assisted electron injection.

### Electron injection condition in presence of a nanoparticle

Even though the PIC simulations can verify the injection scheme by nanoparticle, we develop an analytical formalism to provide an in-depth physical interpretation of the effect of nanoparticle potential on the single particle motion which can lead to injection in the wakefield. The Hamiltonian for electron under the influence of nanoparticle potential, wakefield, and laser field can be written as $H=\sqrt{1+{{\boldsymbol{p}}}^{2}+{{\boldsymbol{a}}}^{2}}-{\varphi }_{wake}(z-{v}_{0}t)-{\varphi }_{nano}(z,t)$, where *H* is normalized by *mc*^2^, ***p*** is the electron kinetic momentum normalized by *mc*, ***a***( = *e****A***/*mc*^2^) is the normalized vector potential of the laser envelope, *v*_0_ is the phase velocity of the plasma wave, *m* is the electron mass, *e* is the electron charge, and *c* is the speed of light in vacuum. For other variables, spatial variables are normalized by plasma wave number *k*_*p*_, time by plasma frequency *ω*_*p*_( = *k*_*p*_*c*), potentials by *mc*^2^/*e*, and velocity *v* by *c*. Since a nanoparticle is very small compared to the laser pulse length, *φ*_*nano*_ is a function of the charge separation, which grows in time as the laser passes over the nanoparticle. Thus, we can further consider *ϕ*_*nano*_(*z*, *t*) = *φ*_*nano*_(*z*)Γ(*t*), where Γ(*t*) is the scale factor to indicate the growing nanoparticle potential. Γ(*t*) varies from 0 to 1, where Γ = 1 for a nanoparticle with all the electrons have been displaced. Henceforth, we adopt the co-moving frame of reference (*ξ* = *z* − *v*_0_*t*, *x*, *y*, *t*) following the laser, in which the Hamiltonian can be expressed as $H=\sqrt{1+{{\boldsymbol{p}}}^{2}+{{\boldsymbol{a}}}^{2}}-{\varphi }_{wake}(\xi )-{\rm{\Gamma }}(t){\phi }_{nano}(\xi +{v}_{0}t)-{v}_{0}{p}_{x}$.

In the absence of a nanoparticle, for a stationary plasma wave in the co-moving frame of reference and thus for a constant Hamiltonian, the injection condition is ${\rm{\Delta }}{\varphi }_{wake} < -\,1$^20^, where ${\rm{\Delta }}{\varphi }_{wake}$is the potential difference experienced by an electron from an initially quiescent state to an injection moment. In the presence of a nanoparticle, however, because the nanoparticle potential, which grows with time and moves with −*v*_0_ in the co-moving frame, the Hamiltonian is no longer constant of motion. We thus define the Hamiltonian difference Δ*H*, induced by the interaction of an electron with the nanoparticle potential in the period from the initial state to the injection moment, and the injection condition should be modified to ${\rm{\Delta }}{\phi }_{wake}+{\rm{\Delta }}H < -\,1$. The explicit form of Δ*H* can be derived using *dH*/*dt* = ∂*H*/∂*t*^11,30^

1 $$\begin{array}{rcl}{\rm{\Delta }}H & = & {\int }^{}\frac{d{\rm{\xi }}}{{v}_{z}-{v}_{0}}\frac{\partial H}{\partial {\rm{t}}}\\  & = & {\int }^{}\frac{-1}{{v}_{z}-{v}_{0}}\frac{\partial {\rm{\Gamma }}(t)}{\partial {\rm{t}}}{{\rm{\phi }}}_{nano}({\rm{\xi }}+{v}_{0}t)d{\rm{\xi }}\\  &  & +{\int }^{}\frac{-1}{{v}_{z}-{v}_{0}}{\rm{\Gamma }}(t)\frac{\partial {\phi }_{nano}(\xi +{v}_{0}t)}{\partial {\rm{t}}}d\xi ,\end{array}$$

where *v*_*z*_ is the longitudinal velocity of the electron. The first term of equation (1) describes the effect of the growing nanoparticle potential as the charge separation increases. If Γ(*t*) rises in a short time, Δ*H* can be simplified to approximately $-{\int }^{}\frac{\partial {\rm{\Gamma }}(t)}{\partial t}{{\rm{\phi }}}_{nano}(z)dt \sim -\,{{\rm{\Delta }}{\rm{\phi }}}_{nano} \sim -\,\langle {v}_{z}\rangle {\rm{\Delta }}{p}_{z,n}$ using *dξ*/(*v*_*z*_ − *v*_0_) = *dz*/*v*_*z*_. Here, *v*_*z*_ is the averaged velocity during the growth of *ϕ*_*nano*_, and ${\rm{\Delta }}{p}_{z,n}$ is the additional momentum obtained from the nanoparticle interaction. As the electron velocity *v*_*z*_ in the front part of the pulse is positive, the first term of equation (1) has a negative value that can relax the injection condition with a positive ${\rm{\Delta }}{p}_{z,n}$. When Γ(*t*) ~ 1, the second term of equation (1) becomes dominant. This term can also be simplified to ${v}_{0}{\int }^{}{E}_{nano,z}({\rm{z}})dt\cong -\,{v}_{0}{\rm{\Delta }}{p}_{z,n}$, where *E*_*nano*,*z*_ is the longitudinal component of the nanoparticle field. A positive ${\rm{\Delta }}{p}_{z,n}$ relaxes the injection condition after full growth (Γ(*t*) ~ 1) of nanoparticle field. In this case, Eq.(1) reduces to ${\rm{\Delta }}H\approx -\,{v}_{0}{\rm{\Delta }}{p}_{z,n}$, and the injection condition can be simply written by ${\rm{\Delta }}{p}_{z,n} > 1+{\rm{\Delta }}{\phi }_{wake}$. Therefore, the momentum gain from the interaction between a nanoparticle field and electron can assist the electron injection into the plasma waves.

We examined the injection condition for electron trajectories from PIC simulations with and without a nanoparticle. The wake potential before interacting with the nanoparticle is represented by a black dashed line. In this particular example, ${{\rm{\Delta }}{\rm{\phi }}}_{wake}=-\,0.4$, electrons cannot be injected without the nanoparticle. To calculate the momentum gain by the nanoparticle field, we tracked the same indexed particles from the simulation performed without the nanoparticle. The black arrow in Fig. 2(c) indicates the momentum gain immediately after the interaction with the nanoparticle field. The momentum gain is ${\rm{\Delta }}{p}_{z,n} \sim \,0.8$, which is sufficient to reach the injection threshold. As shown in the figure, the injection threshold is reached at about z = −20 μm before the electrons reach the back of the plasma wave (z = −25 μm). Thus, the electrons can be injected by obtaining the additional momentum, even in plasma conditions where self-injection cannot be triggered.

The process of momentum gain can be verified by tracking the electron trajectories in space as shown in Fig. 2(d). Considering the laser propagation from left to right, the first electron is initially located on the left (red) and the second on the right side (blue) of the nanoparticle. Both electrons are located about 1-μm away from the center of the nanoparticle. For ease of understanding, we depict the electron trajectories projected in the plane of laser polarization. The electron from the left side moves along an almost straight trajectory starting from an acceleration zone and crossing through a deceleration zone. This electron can be dominantly affected by the growth of the nanoparticle field, as depicted in the first term of Eq.(1). The electrons experience a negative Δ*H* (or increasing momentum) with an increasing nanoparticle field and can be injected. In contrast, the electron starting from the right side, following a circular motion around the nanoparticle similar to slingshot motion, can be injected, as the electron is first attracted toward the nanoparticle (shown in Fig. 2(d)). In this case, the electron obtains additional longitudinal momentum by reducing the effects of the deceleration part of the nanoparticle field. Both cases indicate that electrons closely located around the nanoparticle can be injected by obtaining additional longitudinal momentum.

### Effects of nanoparticle’s material density

**Figure 3:**
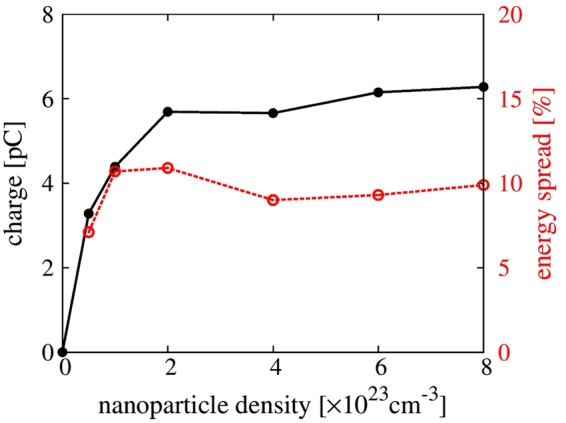
Dependence of injected electron charge and energy spread on electron density due to the nanoparticle based on a series of 3D simulations.

The electron beam charge can be increased by increasing the material density of the nanoparticles which, consequently, increases the strength of the nanoparticle field. To understand the effect of nanoparticle density, another series of 3D PIC simulations with different nanoparticle densities were performed using identical laser and plasma parameters as in Fig. 2. We assume that the ion density of the nanoparticle is proportional to the material density at a given incident laser intensity. Figure 3 presents the results where the laser propagates 0.75 mm. The injected charge increases with the electron density of the nanoparticle (black circles in Fig. 3); however, it starts to saturate when the nanoparticle density reaches the saturation density *n*_*nsat*_ = 1 × 10^23^ cm^−3^. This tendency is similar to the trend exhibited by the energy spread (red circles in Fig. 3). Within the saturation density, the charge follows ${Q}_{beam}\propto {n}_{n}{r}_{n}^{3}$. The charge saturation can be understood from the saturation of the nanoparticle field. In the nanoparticle field, two forces act on the electrons in the nanoparticle: the repelling ponderomotive force by the laser pulse and the attracting force exerted by the positively charged nanoparticle. The movement of electrons is determined by the sum of the ponderomotive potential and the nanoparticle electric potential. When the ponderomotive potential is balanced by the nanoparticle potential, the nanoparticle field cannot grow further because the repelling of electrons is no longer possible and the injected charge can reach saturation. Therefore, the maximum injected charge is controllable by changing the material density of the nanoparticle within the saturation density.

### Effects of the number of nanoparticles

**Figure 4:**
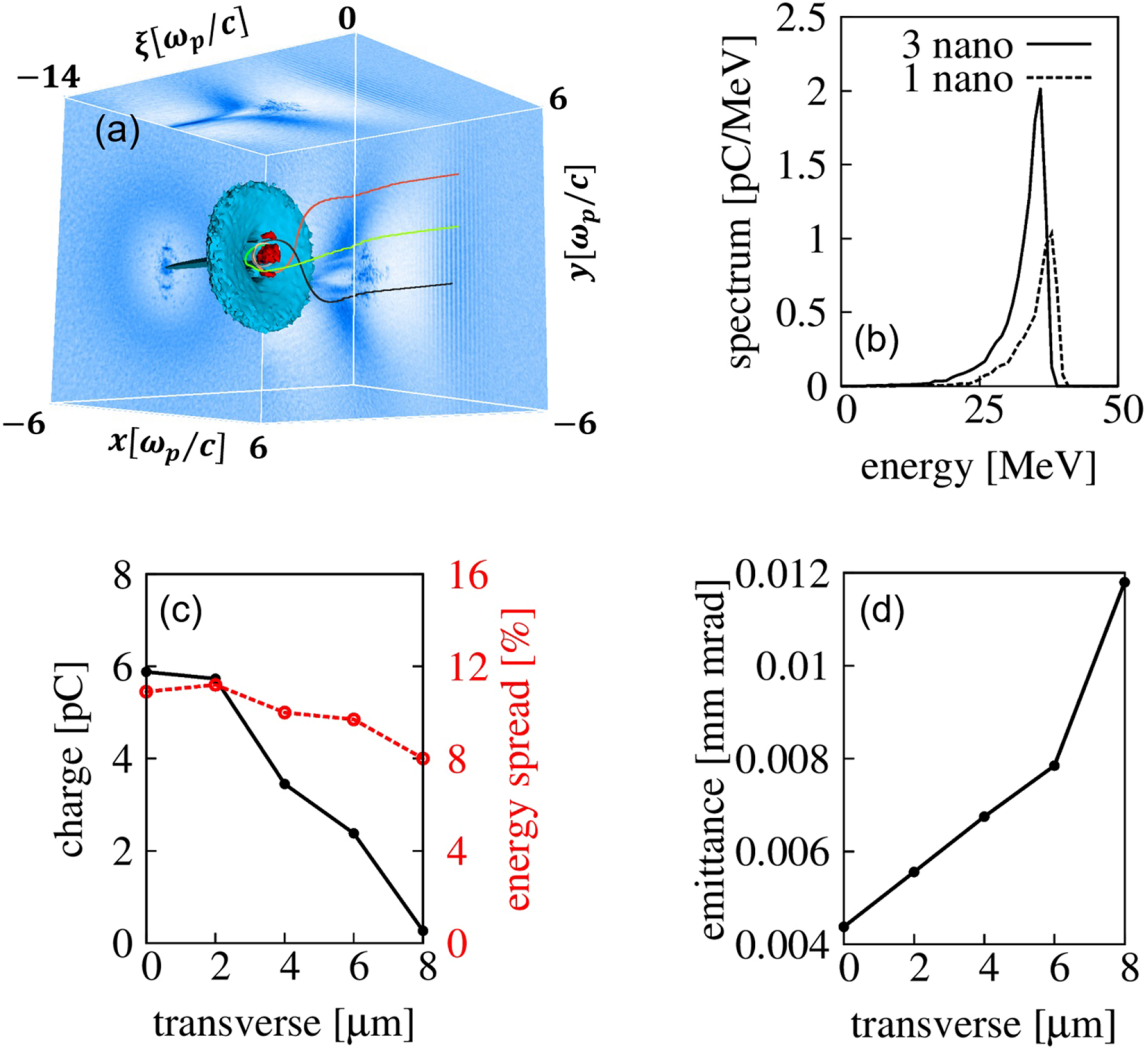
The effect of nanoparticle number and loading position. (**a**) Electron density distribution of three nanoparticles. Three colored lines show the electron trajectories of the nanoparticles. (**b**) Comparison of electron spectra with one (dashed line) and three nanoparticles (solid line) after 0.75 mm propagation in plasma. (**c**) Injected electron charge and energy spread, (**d**) emittance by a single nanoparticle at different loading positions in the transverse direction from the laser axis.

We further investigated the beam loading effect determined by the presence of multiple nanoparticles. In the 3D PIC simulations, three nanoparticles were loaded at different transverse positions, equally spaced by 4 μm, equivalent to half the wakefield radius, and inserted at the same longitudinal position. As shown in Fig. 4a, the three individual nanoparticles induce three different injections. The energy spread does not grow with the number of nanoparticles, as shown in Fig. 4b. The case of three nanoparticles has an energy spread of 12%, almost the same as the one-nanoparticle case of 11%, because the energy spread of the electron beam is insensitive to the transverse nanoparticle position, as indicated in Fig. 4c. As the number of nanoparticles increases from 1 to 3, the injected charge increases from 6 to 11 pC. The total charge is not proportional to the number of nanoparticles because the amount of injected charge depends on the transverse nanoparticle position, as shown in Fig. 4c. Farther from the laser axis, the injected beam charge decreases because the strength of the wakefield at the off-axis position is weaker. In a separate simulation performed for the case of three nanoparticles placed with 10 μm separation along the longitudinal direction, we could still obtain the increased charge without sacrificing the energy spread.

The transverse location of nanoparticles can also affect the beam emittance due to the different transverse momentum. Figure 4d shows that the beam emittance increases with the transverse position of the nanoparticle. Since the longitudinal momentums are similar for all cases, the transverse momentum is the main factor to increase the emittance. Thus, the transverse insertion position of the nanoparticle should be controlled within a few microns to obtain the lowest beam emittance.

### 5.1-GeV electron beam with a 0.8-% energy spread driven by a 0.5 PW laser pulse

**Figure 5:**
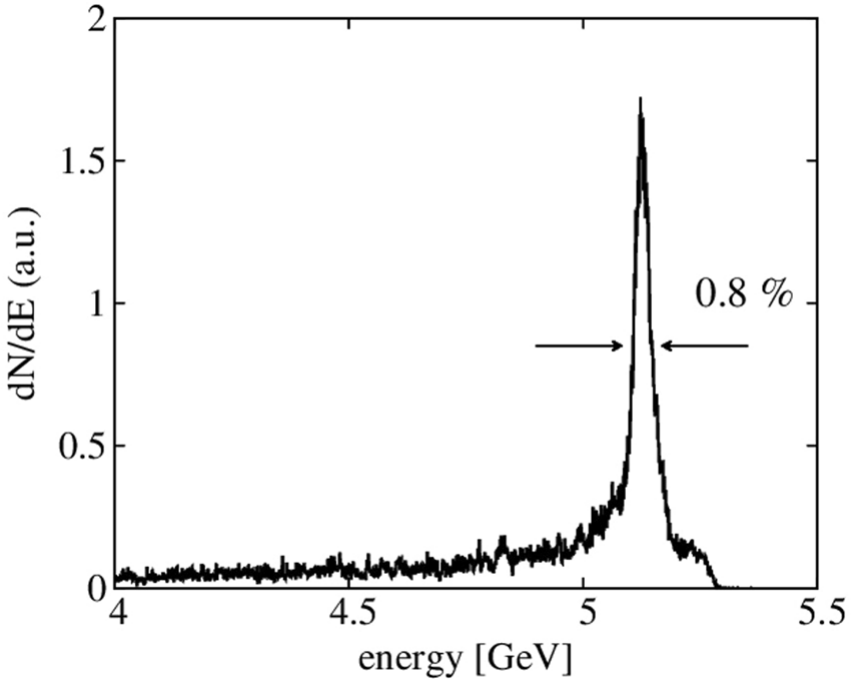
Electron energy spectrum from 2D PIC simulation with a 0.5 PW laser pulse propagating through a 27-cm plasma channel.

Our simulations also suggest that the nanoparticle-induced electron injection can lead to a multi-GeV electron bunch with a significantly lower (~1%) energy spread. To investigate a multi-GeV electron beam, we conducted 2D PIC simulations because of the high computational cost required for propagations tens of centimeters long. As the nanoparticle induces a localized electron injection where the laser wakefield is almost linear, the energy spread of the electron beam can decrease with the electron energy by the acceleration. The laser and plasma parameters are selected to prohibit the electrons self-injection as well as serious laser evolution. The energy loss rate of the laser is around 1.5%/cm, and *a*_0_ reduces to 2.55 (initially 3.5) after 27 cm propagation. Details of the simulation parameters are given in the method section. As shown in Fig. 5, we obtained an electron beam with an energy of 5.1 GeV and small energy spread of 0.8% in FWHM from a 27-cm-long plasma medium.

The main reason for narrow energy spread is a relative size of nanoparticle field to the wakefield. Since the electron injection occurs by the nanoparticle potential, the dominant region of electron injection can be determined by the effective size of the nanoparticle field. Thus, when the wakefield is quasi-linear and the nanoparticle field is given, the energy spread is proportional to the background plasma density. In the simulation, the background plasma density used in 2D is one order smaller than that of the 3D simulation. Thus, it is reasonable to get 1% energy spread, one order of magnitude smaller than in Fig. 2.

## Discussion

In experiments, the precise control of the position and the number of nanoparticles can be difficult. If nanoparticles are distributed over a certain volume, the electron injection also can be elongated along the nanoparticle distribution. The spatial distribution of nanoparticle insertion may broaden the energy spread of the electron beam. However, we found that the energy spread was not so significant when the longitudinal nanoparticle distribution did not exceed plasma wavelength. From 3D PIC simulations, we obtained the energy spread of 14% when 50 nanoparticles were located within 10 μm with a background plasma density of 7 × 10^18^ *cm*^−3^, corresponding to the plasma wavelength of 12 μm, while it was 11% with a single nanoparticle placed on axis. Thus, in order to avoid any extra electron energy broadening due to a nanoparticle distribution, nanoparticles should be inserted to a spot smaller than the plasma wavelength.

The nanoparticle insertion method for LWFA looks similar with the cluster target^31,32^ where nano-scaled solids are mixed with a background gas. However, the acceleration mechanism is entirely different, where the either the dominant mechanism for acceleration is direct acceleration or the injection mechanism in LWFA is ionization injection. Normally to form clusters high gas pressure is required, thus the cluster method is not suitable for a low-density plasma. In addition, since the nanoparticle insertion method is independent of the background density, it does not have any constraint on the gas density.

**Figure 6:**
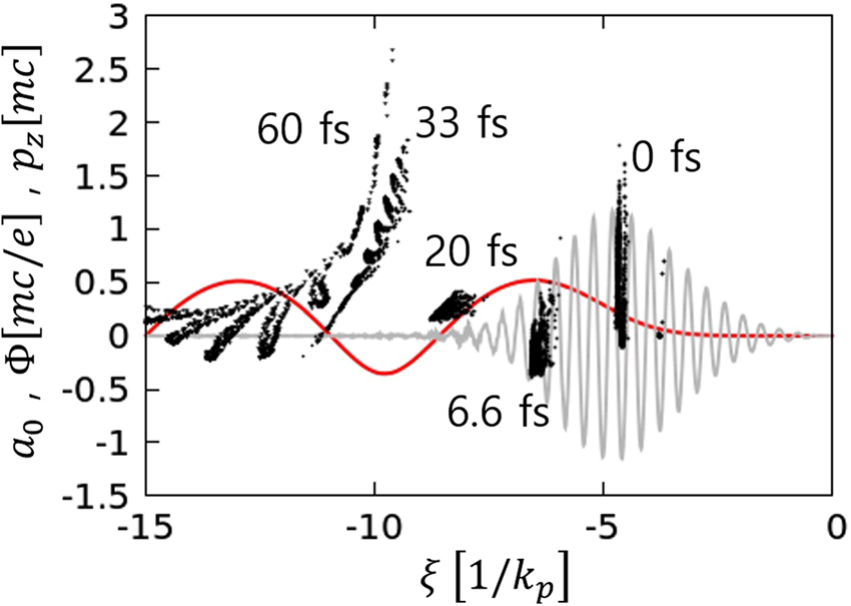
The electrons’ phase space (black dots), laser profile (gray line), and wake potential (red line) in the laser co-propagating coordinate. The time zero is set to the peak of the laser pulse. The ADK model has been used for the ionization rate calculation.

A nanoparticle have higher atomic numbers than hydrogen or helium, the ionization injection^20^ may occur by ionizing inner-shell electrons of the nanoparticle at the peak of a laser pulse, however we didn’t observe any ionization injection in our set-up. To verify this, we performed another set of 3D simulations with identical simulation parameters as in Fig. 2, but with nanoparticle density same as background density, so that the nanoparticle field effects can be ignored. For a nanoparticle, C4+ is loaded with the radius of 50 nm. The evolution of laser pulse is not serious; *a*_0_ slightly increases to 1.2 at the nanoparticle position, where 80% of C5+ and 5% of C6+ can be created at the peak of laser following ADK calculation^33^. The difficulty of ionization injection is presented in Fig. 6. Most of the electrons comes from C5+ and C6+ at the peak of the laser, and at that moment the intrinsic potential difference is around 0.7 that is not sufficient for the injection. Therefore, in this paper, the electron injection occurred only by nanoparticle insertion.

## Conclusion

We investigated the nanoparticle-assisted electron injection method by performing PIC simulations and formulating an analytical model for the injection characteristics. The nanoparticle field reduces the threshold of electron injection as a result of the additional electron momentum. As the nanoparticle creates a very localized strong electrostatic field, it can induce a robust and localized electron injection, which results in an small 0.8% energy spread for a 5-GeV electron beam in a 2D PIC simulation. We found that the electron injection is controllable by tuning the nanoparticle parameters such as size, density, position, and number. Our scheme can be applied to obtain multi-GeV electron beams with low energy spreads below 1%, which can provide an exciting perspective to develop an LWFA-based compact X-ray free electron laser and monochromatic Compton γ-ray sources.

## Methods

In 3D PIC simulations, we used a Gaussian laser pulse linearly polarized in the *y*-direction with a peak power of 8 TW, wavelength of 0.8 µm, normalized vector potential *a*_0_ = 1.0, spot size *W*_0_ = 17.6 μm in full-width at half-maximum (FWHM), and temporal duration *τ*_0_ = 16fs in FWHM. The plasma medium has a 100-μm linear density ramp leading to a homogeneous plasma density n_0_ = 7.0 × 10^18^ cm^−3^. A nanoparticle was inserted at 400 μm inside the plasma and had an electron density of 2 × 10^23^ cm^−3^(≈115n_c_, where n_c_ is the critical density) and diameter of 100 nm. The nanoparticle was treated as a nanometer-scale plasma, which is valid when the laser pulse is sufficiently strong to partially or fully ionize a solid material^34^. The grids sizes were Δz = *λ*_*L*_/40, Δx = Δy = *λ*_*L*_/5; the corresponding grids were 1500 × 313 × 313 with two macro-particles per cell for the ambient plasma. Each cell in the nanoparticle contained 8000 macro-particles for electrons and ions. Even though Δx and Δy were larger than the nanoparticle size, we confined the macro-particles within the dimensions of the nanoparticle. Other simulation with half of the grid size (transversely resolving the nanoparticle with two cells) also gave the same results. Empirically, the grid size was sensitive in the longitudinal direction.

In 2D PIC simulation, the simulation parameters used were a normalized vector potential *a*_0_ = 3.5, FWHM spot size *W*_0_ = 40 μm, and FWHM pulse duration of 30 fs corresponding to a laser power of 500 TW with the same grid resolutions as the 3D simulations. The plasma density was *n*_0_ = 1 × 10^17^ cm^−3^. To ensure laser guiding, we set a transverse parabolic plasma channel with a density profile defined as ${\rm{n}}={n}_{0}\,[1+{r}^{2}/3.7{{W}_{0}}^{2}]$^29^. The nanoparticle parameters were *n*_*n*_ = 5×10^21^ cm^−3^ and *r*_*n*_ = 40nm. A nanoparticle in 2D is considered as a wire, not a sphere as in 3D. At the same condition of the nanoparticle, thus, the 2D nanoparticle field is stronger in space than that of the 3D. Considering this dimension effect, we chose the 2D nanoparticle density and size to give a similar nanoparticle field profile in 3D.
